# The quality of public sources of drinking water in oil-bearing communities in the Niger Delta region of Nigeria

**DOI:** 10.12688/aasopenres.12964.1

**Published:** 2019-07-10

**Authors:** Omosivie Maduka, Benson Ephraim-Emmanuel

**Affiliations:** 1 Department of Preventive and Social Medicine, University of Port Harcourt, Port Harcourt, Nigeria

**Keywords:** Drinking water, oil-bearing community, Niger Delta

## Abstract

**Background: **Studies carried out in the Niger Delta region of Nigeria have demonstrated a link between oil exploration and poor-quality drinking water. However, many of these studies have been limited by small coverage and focus on few parameters. This study thus aimed at a comprehensive assessment of the quality of public sources of drinking water in three gas flaring and three non-gas flaring communities in the Niger Delta region of Nigeria.

**Methods: **A total of 13 samples were collected from the major sources of drinking water in six communities in Rivers, Bayelsa and Delta States, Nigeria. These were stored and transported in line with International standards to a certified environmental laboratory where physical, chemical, bacteriological and petro-chemical assessments were conducted for 27 parameters.

**Results: **Some samples had a pH below the normal range for drinking water, with median pH value of 4.63. All chemical parameters assessed fell below the normal acceptable range with exception of magnesium which exceeded the acceptable range. There were11 samples (91.7%) with microbial contamination; total and faecal coliform demonstrated at values ranging between 15 and 90 most probably number (MPN)/100 ml for total coliform and 9 to 23 MPN/100 ml for faecal coliforms. Oil, grease and total petroleum hydrocarbons (TPH) were identified in water samples from all communities. Values for oil and grease ranged between <0.001 and 0.015 mg/l, while TPH values were between <0.001 and 0.046 mg/l. There was no significant difference between median values in gas flaring and non-gas flaring communities.

**Conclusion: **Distortion of physico-chemical properties, and hydrocarbon and faecal contamination of drinking water are a major challenge in oil-bearing communities in the Niger Delta region of Nigeria irrespective of gas flaring status. This calls for urgent interventions to improve the quality of drinking water for the people of the Niger Delta.

## Introduction

Nigeria is Africa’s largest producer of crude oil and the sixth largest producer in the world, ( Egwurugwu *et al*., 2013; Ikeke, 2013; Oni & Oyewo, 2011) with a capacity to produce approximately 2.5 million barrels per day.( Nigerian National Petroleum Corportaion, 2015)

A large proportion (97%) of the revenue from foreign trade that comes to Nigeria is from export of crude oil. As much as 20% of the country’s GDP and 65% of the revenue budget is sourced from crude oil. Alongside the vast deposit of crude oil are even more vast deposits of natural gas located in the Niger Delta. ( American Association for the Advancement of Science, 2011; Friends of the Earth Limited, 2004) In spite of this, the country is currently ranked by the World Bank as the ‘poverty capital of the world’ with up to half of its population said to be surviving on less than 1.90 dollars a day. ( Kharas *et al*., 2018)

The nine oil-bearing states in the country are collectively known as the Niger Delta. They are: Abia, Akwa Ibom, Bayelsa, Cross River, Delta, Edo, Imo, Ondo and Rivers States. Together they occupy 7.5% of the land mass of the country and are home to 31 million people speaking 250 different dialects from over 40 ethnic groups and 185 Local Government Areas (LGAs). These states though socially and culturally diverse, share in common the deleterious consequences of crude oil exploration and gas flaring. ( Yakubu, 2017a)

Oil exploration and gas flaring, which are routinely carried out by local and international oil companies in Nigeria, poses a significant hazard to the health of populations exposed to it. It has the potential to pollute the environment, heat up the atmosphere and releases greenhouse gases.( Ite & Ibok, 2013; McMichael *et al*., 2003) The process of oil exploration often involves gas flaring which results in the dissemination of greenhouse gases and other air pollutants such as carbon dioxide (CO _2_), methane (CH _4_), ethane, propane, butane hydrogen sulfide (H _2_S), and nitrous oxide (NO _2_). Greenhouse gases have long been implicated in global warming and climate change. ( Ajugwo, 2013; World Health Organization, 2003a; World Health Organization, 2003b) Apart from the effect on atmospheric temperature, the gases flared serve as pollutants to air and water. Greenhouse gases also precipitate the formation of acid rain. Acid rain runs off into surface water and percolates into ground water, causing a reduction in the pH of water and contamination with pollutants. ( Efe & Mogborukor, 2014; Nwankwo & Ogagarue, 2011) There have also been various incidents of oil spills in several locations in the Niger Delta from accidents during drilling and transportation of crude oil. Oil spills contaminate soil, vegetation and water sources and contribute to reduction in the portability of drinking water through the introduction of total petroleum hydrocarbon (TPH). ( Hagras, 2013; Kponee *et al*., 2015; Taiwo *et al*., 2012; WHO, 2005) Microbial contamination is also a common feature as a result of poor sanitation practices which persists despite the fact that oil-bearing communities generate a lot of revenue for oil exploration companies specifically and the nation generally.

The negative effect of oil exploration and gas flaring on the quality of drinking water in specific locations in the Niger Delta has been documented. Studies carried out in the Niger Delta region of Nigeria and elsewhere have demonstrated changes in the quality of drinking water in comparison to International standards. However, these studies have mostly been conducted in one or two communities in a state with focus on only a few parameters. ( Efe & Mogborukor, 2012; Hagras, 2013; Ite & Ibok, 2013) This study thus aimed at a comprehensive assessment of the quality of public sources of drinking water in three gas flaring and three non-gas flaring communities in three states of the Niger Delta region of Nigeria.

## Methods

### Study background

This research was carried out between April and May 2016 in the Niger Delta region of Nigeria. A cross-sectional study design was employed. Communities that had been host to oil exploration activities with gas flaring for a minimum of ten years were included as well as communities without any history of oil exploration activities. A multistage sampling was used in recruiting the study communities. Purposive sampling of Rivers state, Bayelsa state and Delta state out of the nine states in the Niger Delta region of the country was done because these three states arguably have the highest level of oil exploration activities in the Niger Delta. Secondly, simple random sampling of one LGA per selected state from a sampling frame of all LGAs involved in oil exploration activities including gas flaring. Finally, purposive sampling of two communities in each selected LGA was done based on the presence or absence of oil exploration and gas flaring sites, minimal security risk and geographical accessibility. A total of six communities, Sampou and Nedugo in Bayelsa State, Ibada-Elume and Oton-Yasere in Delta state, and Omerelu and Mbodo-Aluu in Rivers State, were selected. Sampou, Ibada-Elume and Omerelu are communities without any oil exploration or gas flaring activities, while Nedugo, Oton-Yasere and Mbodo-Aluu are communities which have been host to oil exploration and gas flaring activities for the past 10 years.

### Sources of water samples

The required permission to obtain water samples for hydro-chemical analysis was sought from the community leadership during community entry activities. Water was sampled from a minimum of two of the most frequently patronized sources of drinking water for each community as reported by the community leadership. One sample per source of drinking water was collected. A total of 13 samples were collected from the six communities. Water samples were collected for physical, chemical and bacteriological assessments. For physical and chemical analyses, water samples were collected in polysterene bottles. Each bottle was washed and rinsed out several times with water and finally with distilled water before sample collection. Bottle sizes ranged from 0.5 to 1.5 litres. Samples for bacteriological analysis were collected into 200 ml sterilized containers and transported to the laboratory within 72 hours. In the laboratory physical, chemical and bacteriological assessments were carried out as follows: colour, odour, electrical conductivity, pH, taste and turbidity for physical properties; alkalinity, hardness, total dissolved solids (TDS), chromium, arsenic, chlorides, sulphate, nitrates, fluorides, sodium, potassium, calcium, magnesium, bicarbonate, iron, ammonia, phosphate, oil and grease for chemical and hydro-chemical properties and Coliform and *Escherichia coli* populations for biological properties.

### Testing procedures

Various laboratory testing procedures were used. Total dissolved solids (TDS) was analysed using the TDS meter (APHA – 209C method). Tests for ammonia (APHA 4500-NH _3_C method), sulphate (APHA 4500 SO _4_^2-^E method), bicarbonate (APHA 2320B method), phosphate (APHA 4500P-E method), chloride (APHA 4500B method), nitrates (APHA 4500E method) were also done. Hardness of water samples (calcium and magnesium contents) were tested using the Titrimetric method. ( Deshpande, 2012) This method is based on the principle that ethylene di-amine-tetra-acetic acid and its sodium salt (abbreviated EDTA) form a chelated soluble complex when added to a solution of certain metal cations. The concentration (in mg/l) of metals in the water samples was determined using an atomic absorption spectrophotometer. The metals were analyzed using the direct air-acetylene flame method (APHA 3111-B). Oil and grease content was analysed using a spectrophotometric method (AP1 – RP 45, APHA 5520B, EPA 1664). TPH assessment was done using a calibrated HP 5890 gas chromatograph equipped with a capillary column. Commercially available TPH Standard, C8-C40 (Hydrocarbon window defining standards, AccuStandard DRH 0085) was used for the calibration of the Gas Chromatograph. Total coliform count was assessed by most probable number (MPN) presumptive test using the APHA 9221B method, while for faecal coliform MPN confirmatory test using the APHA 9221B method and faecal coliform MPN complete test were done. All QA/QC procedures were strictly adhered to during each process. ( Deshpande, 2012)

### Statistical analysis

The results of the analysis for each water sample was entered into IBM Statistical Package for Social Sciences version 23 and analysed. Descriptive statistics included frequency distribution, median and range of the various parameters. The Mann-Whitney U-test was used to ascertain significant differences between values from gas flaring and non-gas flaring communities for each characteristic of drinking water, with the alpha value set at 0.05. Values were also compared the national standards for drinking water. ( Standards Organization of Nigeria, 2007)

## Results

### Review of water samples

A total of 13 samples of drinking water was collected with seven samples (53.9%) obtained from gas flaring communities. Sample collection occurred mostly under sunny/dry weather conditions (10; 76.9%). Boreholes were the drinking water source in 6 out of 13 samples obtained (46.2%). Algae was observed in 11 out of 13 samples (84.6%) ( Table 1).

**Table 1.** Descriptive characteristics of drinking water samples from oil-bearing communities in three states in the Niger Delta Region of Nigeria.

Characteristics	Frequency (n=13)	Percentage
***State***		
Rivers	4	30.8
Bayelsa	4	30.8
Delta	5	38.5
**Gas Flaring Status**		
Flaring	7	53.9
Non flaring	6	46.1
***Weather***		
Rainy/Wet	3	23.1
Sunny/Dry	10	76.9
***Temperature***		
Cold	1	7.6
Warm	6	46.2
Hot	6	46.2
***Wind***		
Calm	11	84.6
Windy	2	15.4
***Source of water***		
Borehole	6	46.2
Open well	1	7.7
Sanitary well	3	23.1
River	1	7.7
Rain	1	7.7
Stream	1	7.7
***Surface Film?***		
No	12	92.3
Yes	1	7.7
***Algae***		
No	2	15.4
Yes	11	84.6

The median levels of temperature, turbidity, and chlorine in all the states studied, were all above the national standards for drinking water, while values for total dissolved solids, chloride and conductivity were far above national standards. However, pH of all samples was within acceptable limits ( Table 2).

**Table 2.** Atmospheric conditions and physical characteristics of drinking from communities in three states in the Niger Delta Region of Nigeria.

Atmospheric conditions	National standards for drinking water *	Rivers State, median (IQR) [n=4]	Bayelsa State, median (IQR) [n=4]	Delta State, median (IQR) [n=5]
Temperature (°C)	25	29.8 (29.6-30.2)	33.3 (30.6-34.2)	29.7 (29.6-33.2)
Dissolved oxygen	>3	7.05 (6.9-7.1)	6.01 (5.5-6.5)	6.2 (5.3-6.5)
Turbidity (NTU)	5	24.1 (19.3-135.0)	76.9 (59.95-149.0)	111.2 (85.3-159.8)
Conductivity (μS/cm)	1000	27.6 (22.4-151.2)	24.7 (0.00-81.6)	123.0 (94.9-169.5)
pH	6.5-8.5	6.6 (5.1-6.9)	6.9 (6.3-7.0)	6.4 (5.8-7.0)
Chlorine (mg/l)	0.2-0.25	17.4 (11.9-75.7)	44.2 (32.6-83.9)	60.8 (46.5-80.1)
TDS (mg/l)	500	48.9 (13.9-278.5)	29.4 (17.1-69.5)	51.4 (31.5-90.3)
Chloride (mg/l)	250	19.0 (4.2-100.4)	9.5 (5.8-26.9)	20.2 (12.5-35.0)
Total hardness, (mg eq CaCO _3_/l)	150	5.0 (2.2-10.4)	3.2 (2.7-4.6)	4.8 (3.5-6.5)

*Maximum allowed levels by National Standards adapted from: Standards Organization of Nigeria, 2007. IQR = Interquartile range; TDS, total dissolved solids.

A review of the chemical characteristics of drinking water obtained from the three states surveyed showed fluoride, nitrate and sulphate levels to be lower than the National standards. The same observation was made for all cations measured, with the exception of magnesium ( Table 3).

**Table 3.** Chemical characteristics of drinking water samples from communities in three states in the Niger Delta Region of Nigeria.

Chemical constituents	National standards for drinking water *	Rivers State, median (IQR) [n=4]	Bayelsa State, median (IQR) [n=4]	Delta State, median (IQR) [n=5]
***Anions***				
Bicarbonate, (mg/l)		5.5 (3.96-7.3)	10.3 (8.1 -11.2)	6.09 (3.8-7.4)
Alkalinity, (mg/l)	20-200 *	4.5 (3.3-6.0)	8.4 (6.6-9.2)	5.0 (3.1-6.0)
Nitrate, (mg/l)	50	0.17 (0.03-0.42)	0.09 (0.09-0.2)	0.09 (0.05-0.13)
Fluoride, (mg/l)	1.5	0.08 (0.05-0.10)	0.01 (0.003-0.02)	0.01 (0.01-0.03)
Sulphate, (mg/l)	100	5.9 (1.3-31.4)	3.0 (1.8-8.4)	6.3 (1.3-7.8)
Ammonia, (mg/l)		0.05 (0.01-0.25)	0.02 (0.02-0.05)	0.03 (0.01-0.23)
Phosphate, (mg/l)		0.002 (0.001-0.008)	0.002 (0.001-0.004)	0.002 (0.001-0.42)
***Cations***				
Calcium, (mg/l)	200	0.29 (0.10-0.92)	0.05 (0.03-1.02)	0.12 (0.07-0.24)
Sodium, (mg/l)	20	1.45 (1.03-2.77)	0.89 (0.25-1.81)	1.21 (1.20-2.12)
Potassium, (mg/l)	12	0.64 (0.58-0.99)	0.62 (0.31-0.88)	0.56 (0.40-0.83)
Iron, (mg/l)	0.3	0.15 (0.03-0.21)	0.03 (0.02-0.07)	0.08 (0.05-0.15)
Chromium, (mg/l)	0.05	0.001 (0.001 -0.001)	0.001 (0.001 -0.001)	0.001 (0.001 -0.001)
Arsenic, (mg/l)	0.01	0.001 (0.001 -0.001)	0.001 (0.001 -0.001)	0.001 (0.001 -0.001)
Magnesium, (mg/l)	0.2	0.22 (0.08-0.88)	0.09 (0.06-0.93)	1.2 (0.61-1.55)

*Maximum allowed levels by National Standards adapted from: Standards Organization of Nigeria, 2007. IQR = Interquartile range

Assessment of microbiological and hydrocarbon characteristics of drinking water obtained from the three different states surveyed showed that the total coliform, faecal coliform and hydrocarbon levels of these samples where higher than the national standards ( Table 4).

**Table 4.** Microbiological and hydrocarbon characteristics of drinking water samples from communities in three states in the Niger Delta Region of Nigeria.

Variable	National standards for drinking water *	Rivers State, median (IQR) n=4	Bayelsa State, median (IQR) n=4	Delta State, median (IQR) n=5
***Microbiology***				
Total coliform MPN/100 ml	10	38 (12.8-68.0)	51.5 (7.0-86.3)	30.0 (26.5-67.5)
Fecal coliform MPN/100 ml	0	10.0 (9.0-11.8)	9.5 (2.3-19.8)	12.0 (10.5-13.5
***Hydrocarbons***				
Oil and grease, (mg/l)	0	0.002 (0.001-0.01)	0.003 (0.001-0.01)	0.005 (0.001-0.012)
TPH (mg/l)	0	0.02 (0.001-0.03)	0.24 (0.001-0.05)	0.005 (0.001-0.012)

* Maximum allowed levels by National Standards adapted from: Standards Organization of Nigeria, 2007. IQR, interquartile range; TPH, total petroleum hydrocarbons.

All *Underlying data* described above are available from Open Science Framework ( Maduka & Ephraim, 2019)

### Comparison of water samples from gas flaring and non-gas flaring communities

Review of the atmospheric and physical characteristics of drinking water samples from gas flaring and non-gas flaring communities in the three surveyed states showed that there was no statistically significant difference in these characteristics when compared for both the gas flaring and non-gas flaring communities ( Table 5).

**Table 5.** Comparing atmospheric and physical characteristics of drinking water samples from gas flaring and non-gas flaring communities in three states in the Niger Delta Region of Nigeria.

Characteristic	National standards for drinking water	Median (range) for gas flaring host communities	Median (range) for non-gas flaring host communities	Mann-Whitney p-value
Temperature (°C)	25	29.7 (29.6-34.1)	31.4 (29.9-33.2)	0.37
Dissolved oxygen	>3	6.21 (5.59-6.89)	6.45 (5.51-7.08)	0.73
Turbidity (NTU)	5	59.4 (21.6-111.2)	112.65 (62.5-171.8)	0.3
Conductivity	1000	66.2 (25.0-123.6)	88.8 (0.0-191.5)	0.84
pH	6.5-8.5	6.41 (6.4-7.0)	6.85 (5.8-7.00)	1
Chlorine	0.2-0.25	32.3 (21.5-60.8)	61.8 (35.7-84.9)	0.45
TDS (mg/l)	500	51.35 (20.4-77.4)	29.4 (11.7-169.6)	0.73
Chloride (mg/l)	250	20.2 (8.2-29.9)	9.50 (4.4-61.1)	0.84
Total hardness (mg eq CaCO _3_/l)	150	4.99 (4.8-5.0)	3.23 (2.0-9.1)	0.53

TDS, total dissolved solids.

Review of the chemical characteristics of drinking water samples from gas flaring and non-gas flaring communities in the three surveyed states showed that there was no statistically significant difference in these characteristics when comparing the gas flaring and non-gas flaring communities ( Table 6).

**Table 6.** Comparing chemical characteristics of drinking water samples from gas flaring and non-gas flaring communities in three states in the Niger Delta Region of Nigeria.

Chemical characteristic	National standards for drinking water	Gas flaring host communities, median (range)	Non-gas flaring host communities, median (range)	Mann-Whitney p-value
***Anions***				
Bicarbonate, (mg/l)		7.67 (3.79-10.33)	6.22 (4.58-8.1)	0.45
Alkalinity, (mg/l)		6.3 (3.11-8.5)	5.10 (3.75-6.6)	0.45
Nitrate, (mg/l)	50	0.1 (0.09-0.13)	0.09 (0.07-0.30)	0.73
Fluoride, (mg/l)	1.5	0.29 (0.01-0.07)	0.17 (0.009-0.06)	0.63
Sulphate, (mg/l)	100	6.30 (2.56-9.33)	2.37 (0.87-12.08)	0.23
Ammonia, (mg/l)		0.03 (0.02-0.23)	0.02 (0.02-0.13)	0.84
Phosphate, (mg/l)		0.002 (0.001-0.004)	0.002 (0.001-0.03)	0.95
***Cations***				
Calcium, (mg/l)	200	0.15 (0.12-1.1)	0.06 (0.02-0.33)	0.23
Sodium, (mg/l)	20	1.21 (1.10-1.80)	1.21 (0.79-2.99)	1.00
Potassium, (mg/l)	12	0.56 (0.56-0.76)	0.67 (0.48-0.94)	0.53
Iron, (mg/l)	0.3	0.08 (0.08-0.16)	0.03 (0.02-0.15)	0.3
Chromium, (mg/l)	0.05	0.001	0.001	1.00
Arsenic, (mg/l)	0.01	0.001	0.001	1.00
Magnesium, (mg/l)	0.2	0.21 (.05-1.20)	0.67 (0.03-1.89)	0.63

Review of the microbiological and hydro-chemical characteristics of drinking water samples from gas flaring and non-gas flaring communities in the three surveyed states showed that there was no statistically significant difference in these characteristics when compared for both the gas flaring and non-gas flaring communities ( Table 7).

**Table 7.** Comparing microbiological and hydrocarbon characteristics of drinking water samples from gas flaring and non-gas flaring communities in three states in the Niger Delta Region of Nigeria.

Variable	National standards for drinking water	Median (range) for gas flaring host communities	Median (range) for non-gas flaring host communities	Mann-Whitney U p-value
***Microbiology***				
Total Coliform MPN/100ml	10	30.0 (15.0-70.00)	63.5 (24.0-75.0)	0.45
Fecal Coliform MPN/100ml	0	12.0 (9.0-12.0)	10.5 (9.00-12.75	0.95
***Hydrocarbons***				
Oil and Grease, (mg/l)	0	0.002 (0.001-0.005)	0.006 (0.001-0.01)	0.45
TPH (mg/l)	0	0.003	0.03	0.63

MPN, most probable number; TPH, total petroleum hydrocarbons.

## Discussion

The first major finding from this study is that various components of drinking water exceed or are below the required safe standards for drinking water in Nigeria. ( Standards Organization of Nigeria, 2007) Temperature, turbidity, chlorine, dissolved oxygen, magnesium, total coliform, fecal coliform, oil/grease and TPH values were all found to be higher than the national drinking water standard. This finding is corroborated by the research findings of Braide *et al*. (2016) and Ugwoha *et al*. (2017); who reported elevated temperatures in surface and groundwater water samples that were tested. Egwurugwu *et al*. (2013) also reported elevated temperature, dissolved oxygen as well as magnesium levels in water samples obtained from gas flaring areas. The increases in temperature was associated with pollution of the tested samples, which they concluded was probably a result of gas flaring activities. Another study identified distortions in water samples gotten from these gas flaring areas as a direct consequences of the flaring activities which are capable of distorting the chemical and biological composition of the drinking water in affected areas. ( Ugwoha & Omenogor, 2017)

**Figure 1.**
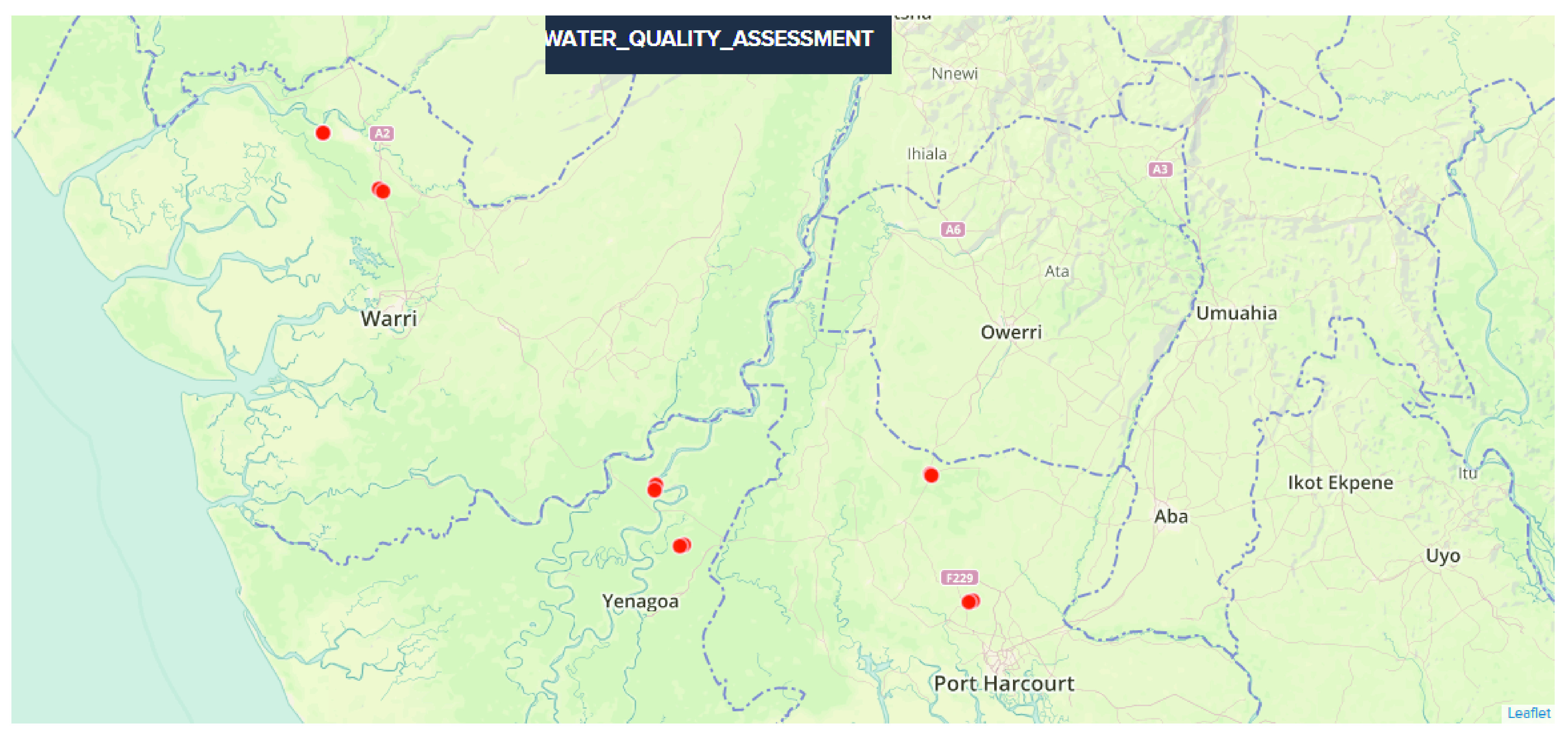
Satellite imaging showing Global Positioning Systems coordinates for locations in the Niger Delta where samples were taken for water analysis.

Also notable in this study was that the pH (of some samples), bicarbonate, iron, fluoride and sulphate values of the tested water samples were found to be lower than the national drinking water standards. These findings are similar to the findings of Dami *et al*. (2012) who reported reduced levels of the above stated parameters. who reported reduced levels of the above stated parameters. These distortions are indicative of the adverse effects of acid rain (which is associated with gas flaring activity) deposition into the water sources from which the samples were gotten and thus increasing the acidity levels of the water beyond the acceptable normal standard for drinking water. ( Idah Seiyaboh, 2018; Ubani & Onyejekwe, 2013)

The need for the availability and consumption of safe drinking water cannot be over-emphasized due to the immense benefits this provides, including elimination of diseases, optimal growth and development, provision of essential biochemical requirements of the body, ensuring public health and safety etc. (Duggal *et al*., 2015). Crude oil exploration in Nigeria which has been incessantly plagued with oil spills mainly as a result of technical errors, pipeline vandalism etc., together with harmful activities such as gas flaring, has adversely affected the health of the populace as well as the biological, economic and socio-cultural elements of the oil-bearing communities of Nigeria ( Efeke *et al*., 2015; Ugwoha & Omenogor, 2017). The activities of crude oil exploration, exploitation and production, including the activity of gas flaring, have been widely reported to negatively impact the normal indices of safe drinking water in these oil-bearing communities as a result of the evaporation, dissolution, oxidization and crystallization of the various components of crude oil whenever an oil spill occurs ( Ekpenyong & Udofia, 2015; Ugwoha & Omenogor, 2017; Yakubu, 2017b)

The findings of this study are suggestive of the negative effects that gas flaring and oil exploration has on the drinking water quality of oil-bearing communities. It is, however, disturbing to note that the differences in the various physical, chemical and biological constituents of tested water samples gotten from gas flaring areas and those from non-gas flaring areas were not significant. This finding is not corroborated by the findings of various studies that reported that the contaminants in the tested water samples were high in the immediate gas flaring areas and reduced in concentration further away from the gas flaring stations. (Amadi, 2014; Ubani & Onyejekwe, 2013) Our findings may be attributable to the weather conditions experienced, especially during the rainy season, which has the capability of influencing the spread of the oil exploration (including gas flaring) contaminants through rain, surface and ground water from gas flaring areas to non-gas flaring areas. It can also be attributable to the spread of these adverse consequences through the dynamic nature of surface and groundwater, especially during the rainy season when rain water levels are increased. (Nkereuwem & Udeme, 2015; Nwankwo & Ogagarue, 2011; Ugwoha & Omenogor, 2017).

In conclusion, the deviation from national standards of drinking water for physico-chemical parameters, as well as hydrocarbon and faecal contamination of drinking water, remain major challenges to the availability of potable drinking water to oil-bearing communities in the Niger Delta region of Nigeria. This calls for urgent interventions to improve the availability of potable drinking water in these oil-bearing communities, such as stricter regulation of gas flaring activities to ensure the health of residents of communities in the Niger Delta region of Nigeria. 

## Data availability

### Underlying data

Open Science Framework: The Quality of Public Sources of Drinking Water in Oil Bearing Communities in the Niger Delta region of Nigeria. https://doi.org/10.17605/OSF.IO/NYMJ8 ( Maduka & Ephraim, 2019).

 Sheet “Water_Quality_Assessment” contains the raw data measured from each sampling area.

Data are available under the terms of the Creative Commons Zero "No rights reserved" data waiver (CC0 1.0 Public domain dedication).

## References

[ref-2] Ajugwo AO: Negative Effects of Gas Flaring: The Nigerian Experience. *J Environ Pollut Hum Heal.* 2013;1(1):6–8. Reference Source

[ref-3] American Association for the Advancement of Science: Geospatial Technologies and Human Rights Project: eyes of Nigeria.2011.

[ref-4] Braide W Nwachukwu J Adeleye SA: Effects of Gas Flaring on the Physicochemical and Microbiological Quality of Water Sources in Egbema, Imo State, Nigeria. *International Letters of Natural Sciences.* 2016;56:7–13. 10.18052/www.scipress.com/ILNS.56.7

[ref-5] Dami A Ayuba HK Amukali O: Effects of Gas Flaring and Oil Spillage on Rainwater Collected forDrinking in Okpai and Beneku, Delta State, Nigeria. *Socialscienceresearch.Org.* 2012;12(13). Reference Source

[ref-6] Deshpande L: Water Quality Analysis Laboratory Methods. *Council of Scientific & Industrial Research, New Delhi, Govt. of India.*New Dehli.2012 Reference Source

[ref-7] Efe S Mogborukor JO: Acid Rain in Niger Delta Region: Implication on Water Resources Quality and Crisis. *AFRREV STECH: An International Journal of Science and Technology.* 2012;1(1):17–46. Reference Source

[ref-8] Efe SI Mogborukor JO: Acid Rain in Niger Delta Region: Implication on Water Resources Quality and Crisis. *AFRREV STECH: An International Journal of Science and Technology.* 2014.

[ref-9] Efeke G Avah I Samuel N: The Effect of Crude Oil Pollution on Water Quality in Niger Delta Region of Bayelsa State. *Nigerian Journal of Health and Allied Research.* 2015;2(1):16–21.

[ref-10] Egwurugwu JN Nwafor A Ezekwe S: Effects of Prolonged Exposure to Gas Flares on the Lipid Profile of Humans in the Niger Delta Region, Nigeria. *Archives of Applied Science Research.* 2013;5(1):98–104.

[ref-11] Ekpenyong N Udofia N: Oil Pollution and Its Impact on Water Quality in Ibeno Community. *Studies in Sociology of Science.* 2015;6(2):8–12. 10.3968/6398

[ref-12] Friends of the Earth Limited: Gas flaring in Nigeria. *Media Report.* 2004;1649 Reference Source

[ref-13] Hagras MA: Water Quality Assessment and Hydrochemical Characteristics of Groundwater in Punjab , Pakistan. *Ijrras.* 2013;16(2):254–262. Reference Source

[ref-14] Idah Seiyaboh E: A Review of Impacts of Gas Flaring on Vegetation and Water Resources in the Niger Delta Region of Nigeria. *International Journal of Economy, Energy and Environment.* 2018;2(4):48–55 10.11648/j.ijeee.20170204.11

[ref-15] Ikeke MO: Thomas Berry’s Idea of Technological Transformation Its Relevance to the Management of Oil Technology in Nigeria’s Niger Delta. *Thought and Practice: A Journal of the Philosophical Association of Kenya (PAK).* 2013;5(1):141–158. Reference Source

[ref-16] Ite AE Ibok UJ: Gas Flaring and Venting Associated with Petroleum Exploration and Production in the Nigeria’s Niger Delta. *American Journal of Environmental Protection.* 2013;1(4):70–77. 10.12691/env-1-4-1

[ref-17] Kharas H Hamel K Hofer M: The start of a new poverty narrative.2018 Reference Source

[ref-18] Kponee KZ Chiger A Kakulu II: Petroleum contaminated water and health symptoms: a cross-sectional pilot study in a rural Nigerian community. *Environ Health.* 2015;14:86. 10.1186/s12940-015-0073-0 26546277 PMC4636824

[ref-19] Maduka O Ephraim B: The Quality of Public Sources of Drinking Water in Oil Bearing Communities in the Niger Delta region of Nigeria.2019 10.17605/OSF.IO/NYMJ8 PMC7721061 33336147

[ref-20] McMichael AJ Campbell-Lendrum DH Corvalan CF: Climate change and human health: Risks and responses.2003 Reference Source

[ref-21] Nigerian National Petroleum Corportaion: Oil Production in Nigeria.2015 Reference Source

[ref-22] Nwankwo CN Ogagarue DO: Effects of gas flaring on surface and ground waters in Delta State Nigeria. *J Geol Min Res.* 2011;3(5):131–136. Reference Source

[ref-23] Oni SI Oyewo MA: Gas Flaring, Transportation and Sustainable Energy Development in the Niger-Delta , Nigeria. *J Hum Ecol.* 2011;33(1):21–28. 10.1080/09709274.2011.11906345

[ref-24] Standards Organization of Nigeria: Nigerian Standard for Drinking Water Quality.2007.

[ref-1] Taiwo AM Olujimi OO Bamgbose O: Surface Water Quality Monitoring in Nigeria: Situational Analysis and Future Management Strategy. In Dr. Voudouris (Ed.):2012;602 10.5772/33720

[ref-25] Ubani E Onyejekwe I: Environmental impact analyses of gas flaring in the Niger delta region of Nigeria. *American Journal of Scientific and Industrial Research.* 2013;4(2):246–252. 10.5251/ajsir.2013.4.2.246.252

[ref-26] Ugwoha E Omenogor EB: Effect of Oil Spillage on Groundwater Quality. *J Environ Stud.* 2017;3(1):1–3. 10.13188/2471-4879.1000019

[ref-27] WHO: Petroleum Products in Drinking-water.2005 Reference Source

[ref-28] World Health Organization: Climate Change and Human Health: risk and responses. (A. J McMichael, D. Campbell-Lendrum, C. Corvalan, K. Ebi A. Githeko, J. Scheraga, & A. Woodward, Eds.) (First). Switzerland: World Health Organization.2003a Reference Source

[ref-29] World Health Organization: Climate Change and Human Health - Risks and Responses Summary. (A. J McMichael, D. Campbell-Lendrum, C. Corvalan, K. Ebi A. Githeko, J. Scheraga, & A. Woodward, Eds.). Switzerland: World Health Organization.2003b Reference Source

[ref-30] Yakubu O: Addressing Environmental Health Problems in Ogoniland through Implementation of United Nations Environment Program Recommendations: Environmental Management Strategies. *Environments.* 2017a;4(2):28 10.3390/environments4020028

[ref-31] Yakubu OH: Particle (Soot) Pollution in Port Harcourt Rivers State, Nigeria—Double Air Pollution Burden? Understanding and Tackling Potential Environmental Public Health Impacts. *Environments.* 2017b;5(1):2 10.3390/environments5010002

