# Supplementary material for: The quality of public sources of drinking water in oil-bearing communities in the Niger Delta region of Nigeria
Source: AAS Open Res. 2019 Jul 10;2:23. [Version 1] doi: 10.12688/aasopenres.12964.1 (PMC7721061; doi:10.12688/aasopenres.12964.1)
Supplement: Peer review history

# Peer review: Reviewer response for version 1

**DOI:** 10.21956/aasopenres.14043.r27998

**Published:** 2020-12-04

**Authors:** Ebenezer Olasunkanmi Dada

**Affiliations:** 1 Department of Cell Biology and Genetics, Environmental Biology Unit, Faculty of Science, University of Lagos, Lagos, Nigeria

This paper assessed the quality of public sources of drinking water in gas flaring and non-gas flaring communities in the Niger Delta region of Nigeria and found the chemical parameters of the sampled water to be generally within the acceptable limits. However, elevated levels of total and faecal coliform, oil, grease, and total petroleum hydrocarbons (TPH) were reported. The Niger Delta region of Nigeria has long been associated with water and soil pollution due to the oil exploration activities in the region. Hence, I consider this study relevant.

 In my estimation, the work is properly designed and accurately reported. The methodology is adequately explained and allows for replication by other researchers. The statistical tool used for data analysis is appropriate and the results are well interpreted. The conclusion is good enough.

 ​​However, I consider the stated aim of the paper: ‘…comprehensive assessment of the quality of public sources of drinking water….’ as an overstatement. The physicochemical and microbial parameters assessed in the study cannot be said to be ‘comprehensive’. There are many more important water quality parameters that were not assessed in the study, including pharmaceuticals, pesticides, herbicides and endocrine disrupting compounds like phthalates. The authors should, in future, be moderate and precise in stating their aim/objectives. In addition, the authors should endeavour to cite more recent literature in their future studies.

Is the work clearly and accurately presented and does it cite the current literature?

Yes

If applicable, is the statistical analysis and its interpretation appropriate?

Yes

Are all the source data underlying the results available to ensure full reproducibility?

Yes

Is the study design appropriate and is the work technically sound?

Yes

Are the conclusions drawn adequately supported by the results?

Yes

Are sufficient details of methods and analysis provided to allow replication by others?

Yes

Reviewer Expertise:

Environmental Biology, Pollution Control, Vermitechnology

I confirm that I have read this submission and believe that I have an appropriate level of expertise to confirm that it is of an acceptable scientific standard.

# Peer review: Reviewer response for version 1

**DOI:** 10.21956/aasopenres.14043.r27742

**Published:** 2020-09-09

**Authors:** Uwemedimbuk Smart Ekanem

**Affiliations:** 1 Department of Community Health, University of Uyo Teaching Hospital, Uyo, Nigeria

The study is well conceptualized. The title aligns with the concept, plan and conduct of the research. The background, introduction and objectives are well stated. The design and methodology is adequate considering the objectives of the study. The analysis and results are well presented and achieved the objectives of the study. The discussion is good.

 However, though the researchers noted with concern that the differences in the various physical, chemical and biological constituents of tested water samples gotten from gas flaring areas and those from non-gas flaring areas, were not statistically significant, they concluded and called for urgent interventions such as stricter regulation of gas flaring activities, when there is no evidence to substantiate this claim from the study. It is appropriate to re-phrase that statement/recommendation.

Is the work clearly and accurately presented and does it cite the current literature?

Yes

If applicable, is the statistical analysis and its interpretation appropriate?

Yes

Are all the source data underlying the results available to ensure full reproducibility?

Yes

Is the study design appropriate and is the work technically sound?

Yes

Are the conclusions drawn adequately supported by the results?

Partly

Are sufficient details of methods and analysis provided to allow replication by others?

Yes

Reviewer Expertise:

Community (Public Health) with interest in Epidemiology and Reproductive health

I confirm that I have read this submission and believe that I have an appropriate level of expertise to confirm that it is of an acceptable scientific standard.
